# Effect of Omicron BA.1-based compared to prototype booster mRNA vaccination on incidence of COVID-19 in the COVAIL trial

**DOI:** 10.1016/j.vaccine.2025.127718

**Published:** 2025-09-09

**Authors:** David J. Diemert, Daniel S. Graciaa, Bo Zhang, Nadine G. Rouphael, Angela R. Branche, Thomas C.S. Martin, Lisa A. Jackson, Rachel M. Presti, Satoshi Kamidani, Siham M. Mahgoub, Tara M. Babu, Craig A. Magaret, Viviana Simon, Harm van Bakel, Paul C. Roberts, John H. Beigel, Peter B. Gilbert, Dean Follmann, for the Coronavirus Variant Immunologic Landscape Trial (COVAIL) Study Team

**Affiliations:** a Department of Medicine, School of Medicine and Health Sciences, The George Washington University, Washington, DC, USA; b Hope Clinic of the Emory Vaccine Center, Division of Infectious Diseases, Emory University School of Medicine, Decatur, GA, USA; c Vaccine and Infectious Disease Division, Fred Hutchinson Cancer Center, Seattle, Washington, USA; d Vaccine and Treatment Evaluation Unit, University of Rochester, Rochester, New York, USA; e University of California, San Diego, La Jolla, California, USA; f Kaiser Permanente Washington Health Research Institute, Seattle, Washington, USA; g Department of Medicine, Washington University School of Medicine, St Louis, MO, USA; h Center for Childhood Infections and Vaccines, Children’s Healthcare of Atlanta, GA, USA; i Department of Pediatrics, Emory University, Atlanta, GA, USA; j Howard University College of Medicine, Howard University Hospital, Washington, DC, USA; k Division of Allergy and Infectious Diseases, Department of Medicine, University of Washington, Seattle, Washington, USA; l Department of Microbiology, Icahn School of Medicine at Mount Sinai, NY, New York, USA; m Center for Vaccine Research and Pandemic Preparedness (C-VaRPP), Icahn School of Medicine at Mount Sinai, New York, NY, USA; n Department of Pathology, Molecular and Cell Based Medicine, Icahn School of Medicine at Mount Sinai, New York, NY, USA; o Division of Infectious Diseases, Department of Medicine, Icahn School of Medicine at Mount Sinai, New York, NY, USA; p The Global Health and Emerging Pathogens Institute, Icahn School of Medicine at Mount Sinai, New York, NY, USA; q Department of Genetics and Genomic Sciences, Icahn School of Medicine at Mount Sinai, New York, NY, USA; r Department of Artificial Intelligence and Human Health, Icahn School of Medicine at Mount Sinai, New York, NY, USA; s Icahn Genomics Institute, Icahn School of Medicine at Mount Sinai, New York, NY, USA; t Division of Microbiology and Infectious Diseases, National Institute of Allergy and Infectious Diseases, National Institutes of Health, Bethesda, MD, USA; u Public Health Sciences Division, Fred Hutchinson Cancer Center, Seattle, Washington, USA; v Department of Biostatistics, School of Public Health, University of Washington, Seattle, Washington, USA; w Biostatistics Research Branch, National Institute of Allergy and Infectious Diseases, National Institutes of Health, Bethesda, MD, USA

**Keywords:** SARS-CoV-2, COVID-19, mRNA vaccine, Variant, Vaccine efficacy

## Abstract

**Background:**

Covid-19 vaccines are updated to match circulating strains based on reasoning that better strain-matched immunogenicity should provide better protection. Randomized evidence with disease endpoints to support strain matching is lacking. We evaluated COVID-19 incidence among adults randomized to a second booster of Prototype or Omicron-based vaccines.

**Methods:**

COVAIL was a four-stage Phase 2 clinical trial; results from Stages 1 (mRNA-1273 [Moderna]) and 2 (BNT162b2 [Pfizer/BioNTech]) are described here. Adults who had received a primary series and one booster of an authorized COVID-19 vaccine were eligible. Participants received one dose of either Prototype vaccine or a monovalent or bivalent Omicron BA.1 vaccine. SARS-CoV-2 neutralization titers (ID_50_) were measured pre- and post-vaccination. Covariate-adjusted cumulative COVID-19 incidence and Cox regression analyses were conducted separately for each stage.

**Results:**

706 participants with pre- and day 15 post-vaccination ID_50_ titers (*n* = 503 in Stage 1, *n* = 203 in Stage 2) were included. Within stages, participant characteristics and baseline ID_50_ titers were similar between Prototype and Omicron-based arms. There was no difference in cumulative COVID-19 incidence for Prototype vs. Omicron-based vaccine in Stage 1 (RR 1.04, 95 % CI 0.73–1.48), while incidence was higher among Prototype recipients in Stage 2 (RR 2.56, 1.44–4.52). Cox regression analysis showed no difference in Stage 1 (HR 1.04, 0.68–1.58), but higher incidence for Prototype recipients in Stage 2 (HR 2.95, 1.52–5.72).

**Conclusions:**

Omicron-based vaccines as second boosters were more protective against COVID-19 relative to Prototype among those receiving BNT162b2 but not mRNA-1273. Differences between stages such as force of infection, antigen matching, and vaccine differences may explain this finding.

**ClinicalTrials.gov Registry Number:**

NCT05289037

## 1. Introduction

Among multiple SARS-CoV-2 vaccines developed using different platform technologies, the two approved ones using the mRNA platform – mRNA-1273 (Moderna) and BNT162b2 (Pfizer/BioNTech) – have shown excellent induction of neutralizing antibodies and protection against severe disease/death [1,2]. However, despite high levels of protection with ancestral-strain vaccines early in the pandemic, waning immunity and emerging variants led to immune escape and successive waves of infection and disease [3,4]. Accordingly, COVID-19 vaccines have been progressively updated to more closely match the spike sequence of circulating viral variant(s), first Omicron BA.4/5 in 2022, then Omicron XBB.1.5 in 2023. In June 2024, the Food and Drug Administration recommended that vaccines be targeted against the KP.2 strain (JN.1 Omicron lineage) [5]. Although logical, evidence from randomized clinical trials showing improved protection with strain matching is lacking.

The Coronavirus Variant Immunologic Landscape Trial (COVAIL) was a Phase 2, open-label, randomized clinical trial whose primary objective was to evaluate humoral immune responses to candidate SARS-CoV-2 variant vaccines in previously vaccinated adults. We previously reported that a boost with an Omicron-based (vs. Prototype) vaccine may provide a serologic advantage against Omicron [6]. Improved understanding of variant vaccine protection relative to previous versions may inform future vaccine development.

Here we compared COVID-19 incidence among participants randomized to receive a second booster dose of monovalent Prototype (ancestral) vs. an Omicron-based (monovalent or bivalent) mRNA vaccine.

## 2. Methods

### 2.1. Study design

COVAIL was an open-label, randomized, Phase 2 trial performed at 22 US sites [6,7]. It was conducted in four sequentially-enrolled stages. This manuscript describes results from participants enrolled in Stages 1 and 2 who received a single dose of the monovalent Prototype (ancestral) mRNA vaccine or a monovalent or bivalent vaccine containing mRNA encoding Omicron BA.1 spike (Table 1). Bivalent vaccines contained mRNA encoding Omicron BA.1 spike combined with mRNA encoding Prototype, Beta (B.1.351), or Delta (B.1.1.617.2) spike. Participants were enrolled between March and May 2022 (Stage 1) or in May 2022 (Stage 2). Eligible participants were healthy adults ≥18 years old, with or without history of prior SARS-CoV-2 infection who had received a primary vaccine series and a single booster dose of an approved or authorized Prototype COVID-19 vaccine. The most recent vaccine dose, and/or prior infection, must have occurred ≥16 weeks before randomization. Assessment of health was determined by medical history, targeted physical examination and investigator clinical judgement. Individuals with unstable chronic medical conditions, advanced liver or kidney disease, or a diagnosis of an immunocompromising condition, were excluded. Complete eligibility criteria are described at clinicaltrials.gov (NCT05289037).

In total, 914 participants (Stage 1: 602; Stage 2: 312) were stratified by age (18–64 vs. ≥65 years) and reported history of SARS-CoV-2 infection, and randomly assigned to each arm of the respective Stage in equal allocations. In Stage 1, 503 received a one-dose Prototype or Omicron-based vaccine, had no eligibility violations or missing Day 1 (D1) or D15 BA.1 neutralization titers, and were included in the cumulative incidence analyses vs. 203 in Stage 2.

COVID-19 cases were detected through active surveillance for symptoms compatible with COVID-19 at clinic visits on D15, D29, and 3-, 6-, 9-, and 12-months post-vaccination, and collection of nasal swabs for nucleic acid amplification testing (NAAT) if symptomatic and outside-study testing had not been performed; and by passive detection, wherein participants were asked to report symptoms or positive external rapid antigen or NAAT tests. For all confirmed COVID-19 cases, an additional nasal swab was collected for viral sequencing. Among symptomatic COVID-19 endpoints detected across mRNA arms, 97.7 % met the CDC clinical criteria [8] and supportive laboratory criteria, while 89.7 % met COVE trial clinical criteria [9] and supportive laboratory criteria.

The trial was reviewed and approved by the Advarra central institutional review board and overseen by an independent Data and Safety Monitoring Board. Written informed consent was obtained from all study volunteers. The trial was sponsored and funded by the National Institutes of Health (NIH).

### 2.2. Study vaccines

Vaccines for Stage 1 were provided by Moderna (Cambridge, MA). Each dose contained 50 μg mRNA, with the bivalent products (Omicron BA.1 [B.1.1.529]/Prototype, Omicron BA.1/Beta [B.1.351], and Omicron BA.1/Delta [B.1.1.617.2]) containing 25 μg of each component. For Stage 2, vaccines were provided by Pfizer/BioNTech. Each dose contained 30 μg mRNA, with the bivalent products (Omicron BA.1 [B.1.1.529]/Prototype and Omicron BA.1/Beta [B.1.351]) containing 15 μg of each component.

### 2.3. Study outcomes

This work addresses the exploratory objective of evaluating breakthrough COVID-19 incidence within each stage following receipt of study vaccine and comparing it between randomized vaccine arms, accounting for history of prior COVID-19 and evaluating humoral immune responses and variant spike lineages.

### 2.4. Immunogenicity assays

Serum SARS-CoV-2 50 % inhibitory dilution neutralization titers (ID_50_) were measured using pseudotyped lentiviruses presenting SARS-CoV-2 spike mutations for D614G (Wuhan-1 strain harboring the D614G mutation), Omicron BA.1 (B.1.1), Omicron BA.4/5, Beta (B.1.351), and Delta (B.1.617.2) [6,10]. An electrochemiluminescence immunoassay (Elecsys Anti-SARS-CoV-2 N, Roche) was used to detect baseline anti-nucleocapsid (anti-N) antibodies [11].

### 2.5. Viral sequencing

RNA was extracted from nasopharyngeal swabs; SARS-CoV-2 levels were quantified; and viral genomes were amplified, sequenced, and mapped to major lineages as described in the Supplementary Material.

### 2.6. Statistical analysis

Since mRNA-1273 and BNT1262b2 were evaluated in different COVAIL stages, analyses were performed separately for each. Prior infection was defined as a self-reported positive rapid antigen or NAAT, or by positive anti-N antibody testing at baseline. A force of infection (FOI) score was calculated for each participant as the average of daily COVID-19 incidence rates in the participant’s state (or the District of Columbia) from the Coronavirus Resource Center’s database hosted by Johns Hopkins University during their approximately 6-month follow-up, scaled at the end to have a mean of zero and standard deviation of one (see the Statistical Analysis Plan published with [12] and recapitulated in the Supplementary Materials of the current article for details on calculation of this score). A baseline risk score was calculated using ensemble machine learning (superlearner) based on the following baseline demographic input variables: age in years, indicator of age ≥ 65 years, sex assigned at birth, race, ethnicity, the number of days from last prior vaccination until enrollment, an indicator that the number of days from last prior vaccination until enrollment was greater than the median value, and the types of vaccine received before enrollment in the COVAIL trial (i.e., the primary series vaccine and the first booster vaccine). The baseline risk score is defined as the logit of the predicted probability of COVID-19 from the superlearner model, where this logit-predicted outcome is scaled to have a mean of zero and standard deviation of one. Baseline characteristics were compared using a chi-squared test (categorical variables) or a Student’s *t*-test (continuous variables).

SARS-CoV-2 pseudovirus neutralization titers, as well as a maximal signal diversity weighted (MDW) average of antibody log_10_ ID_50_ values against D614G, Delta, Beta, BA.1 and BA.4/BA.5 variants, were summarized by group at baseline (pre-study vaccination). Comparisons between mean neutralization titers (log_10_ scale) were made using Student’s t-test.

Covariate-adjusted study time-based cumulative incidence analyses and calendar time-based Cox regression analyses were conducted separately for each stage to assess and compare COVID-19 incidence over time between Prototype and Omicron-based vaccines. Cumulative incidence curves were estimated counting from D1 through 188 days post-Day 15. Covariate-adjusted cumulative incidence ratios were also estimated to characterize the relative vaccine efficacy between Prototype and Omicron-based vaccines. To improve precision, all cumulative incidence analyses were further adjusted for participant baseline factors predictive of COVID-19 that may have caused accidental confounding despite randomization, including prior infection status, FOI score, and risk score.

Calendar-time Cox regression analyses were also conducted to estimate the hazard ratios between Prototype and Omicron-based vaccines. Cox regression analyses adjusted for the risk score and were stratified by a participant’s prior infection status so that participants with different baseline infection status may have had different baseline hazards. Additional analyses were conducted separately after dividing COVID-19 endpoints into booster-proximal (i.e., 7 to 91 days post-D15) and booster-distal (92 to 188 days post-D15) cases.

Cumulative incidence-based and Cox regression analyses are complementary. The former provide estimates of the absolute risk of COVID-19 for Prototype and Omicron-based vaccine groups over time and compare this risk between groups with minimal modeling assumptions. The latter provide estimates of ratios (Omicron-based/Prototype) of the instantaneous risk of COVID-19 and thus have interpretation in terms of immediate-term relative risk of acquiring COVID-19. Being calendar time-based, Cox analyses have the advantage of flexibly accounting for underlying epidemiological trends in COVID-19 incidence.

All comparative analyses, including cumulative incidence-based and Cox regression-based analyses and comparative immunogenicity analyses, further excluded early infections, defined as endpoints that occurred prior to 7 days post-D15. All analyses were pre-specified in the Statistical Analysis Plan (Supplementary Material). All statistical analyses were conducted using R v.4.2.2 or higher. Covariate-adjusted cumulative incidence analyses were performed using the *CFsurvival* R package [13].

## 3. Results

### 3.1. Study population

Seven hundred and six participants who had previously received a primary COVID-19 vaccination and at least one booster dose (see Supplementary Table 1 for a summary of previous vaccines received for participants in each stage), had no eligibility violations and no missing D1 or D15 BA.1 ID_50_ titers, were enrolled between March 30 and May 6, 2022 for Stage 1 (Moderna; *n* = 503), and between May 12 and 27, 2022 for Stage 2 (Pfizer/BioNTech; *n* = 203), and were randomized to receive one dose of either a monovalent prototype mRNA vaccine or an Omicron-based mRNA vaccine. Of the Stage 1 participants included in the analysis, 97 received Prototype vaccine and 406 Omicron-based, vs. 47 Prototype and 156 Omicron-based in Stage 2 (Table 1).

There were no significant differences in means or frequencies between monovalent Prototype and pooled Omicron-based vaccine arms within each stage with respect to age, proportion > 65 years, race, sex, prior infection status, standardized FOI, or risk score (Table 2). Similarly, geometric mean pseudovirus neutralization ID_50_ values at baseline were similar between within-Stage groups for all tested variants and the MDW average.

Although participants were not randomized between stages, we made comparisons between them. Participants in Stage 1 were slightly older (mean age, 51.9 vs. 50.3 years; *P* = 0.26; with a higher proportion ≥ 65 years, 34.6 % vs. 29.1 %; *P* = 0.19), were less likely to have had a prior infection (21.5 % vs. 34.5 %, *P* < 0.001), and had a significantly higher standardized FOI score (mean 0.58 vs. 0.00, *P* < 0.001).

### 3.2. SARS-CoV-2 neutralization responses

Mean baseline and post-booster neutralization titers (log_10_ scale) at D15 are shown in Table 3, as well as the ratios of geometric means and of geometric mean fold increases to Omicron BA.1 compared to D614G. Baseline log_10_ neutralization titers were similar between the two stages (mean against Omicron BA.1 2.51 for Stage 1 vs. 2.54 for Stage 2, *P* = 0.35). Fold increases in neutralization titer to Omicron BA.1 were higher compared to fold increases to D614G in both stages. In Stage 1, Omicron-based vaccine recipients had a higher relative increase in titer to Omicron BA.1 compared to D614G (ratio of geometric mean fold rise in titer to BA.1 vs. D614G = 2.65) compared to Prototype mRNA vaccine recipients (ratio = 1.57). The same pattern was observed in Stage 2, where Omicron-based vaccine recipients had a higher ratio of geometric mean fold rise in titer to BA.1 compared to D614G (ratio = 2.87) compared to monovalent Prototype vaccine recipients (ratio = 1.38). Similarly, the ratios of BA.1 to D614G geometric mean absolute D15 neutralization titers were higher in Omicron-based vaccine recipients in both stages.

When restricted to participants in whom an incident case of COVID-19 was not detected during study follow-up, the decay in both D614G and Omicron BA.1 neutralization levels from D15 to D188 was similar between Prototype and Omicron-based vaccines in both Stages 1 and 2 (Supplementary Fig. 1).

Post-boost neutralization activity against Omicron BA.4/5 was slightly, but not significantly, higher in Stage 1 Omicron-based vaccine recipients than Stage 2 Omicron-based vaccine recipients, with covariate-adjusted D15 geometric mean ID_50_ titers of 1905 AU/ml and 1660 AU/ml (*P* = 0.51), respectively (Supplementary Table 2).

### 3.3. Incidence of COVID-19 post-booster dose

A total of 194 confirmed cases of COVID-19 occurred from D1 through 188 days after D15 in the Stage 1 and 2 study participants that received either the monovalent Prototype or an Omicron-based mRNA vaccine (155 in Stage 1 participants and 39 in Stage 2 participants) (Supplementary Table 3). In Stage 1, among 400 infection-naïve participants, 138 (34.5 %) acquired COVID-19, including 11 early infections (prior to 7 days post-D15, or between D1 and D22), 78 booster-proximal cases (occurring between 7 and 91 days after D15) and 49 booster-distal cases (between 92 and 188 days after D15), whereas among 103 non-naïve participants, a total of 17 (16.5 %) acquired COVID-19, including 1 early infection, 10 booster-proximal cases and 6 booster-distal cases.

In Stage 2, among 133 infection-naïve participants, a total of 32 (24.1 %) acquired COVID-19, including 2 early infections, 23 booster-proximal cases and 7 booster-distal cases, while among 70 non-naïve participants, a total of 7 (10 %) acquired COVID-19, including 6 booster-proximal and 1 booster-distal cases.

### 3.4. Impact of Prototype vs. Omicron-based mRNA vaccines on incidence of COVID-19

**Fig. 1.**
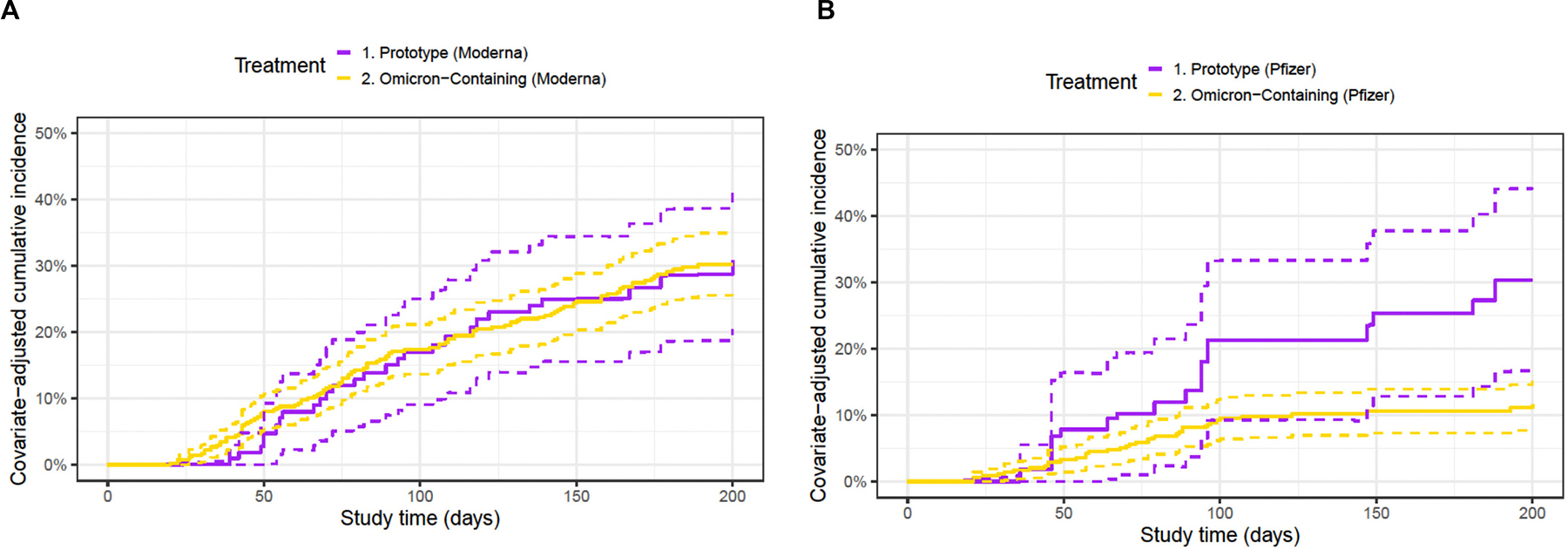
Covariate-adjusted, marginalized cumulative incidence of COVID-19 curves through 188 days post Day 15 visit including COVID-19 endpoints starting 7 days post Day 15 visit for (A) Moderna Stage 1 and (B) Pfizer/BioNTech Stage 2. Purple lines show the curves for the Prototype vaccine, and golden lines for Omicron-based vaccines, comprising (Beta + Omicron BA.1, Delta + Omicron BA.1, Omicron BA.1, Omicron BA.1 + Prototype) pooled for Moderna and (Beta + Omicron BA.1, Omicron BA.1, Omicron BA.1 + Prototype) pooled for Pfizer/BioNTech. Solid lines are point estimates, dashed lines are 95 % pointwise confidence intervals. Study time is days since booster.

**Fig. 2.**
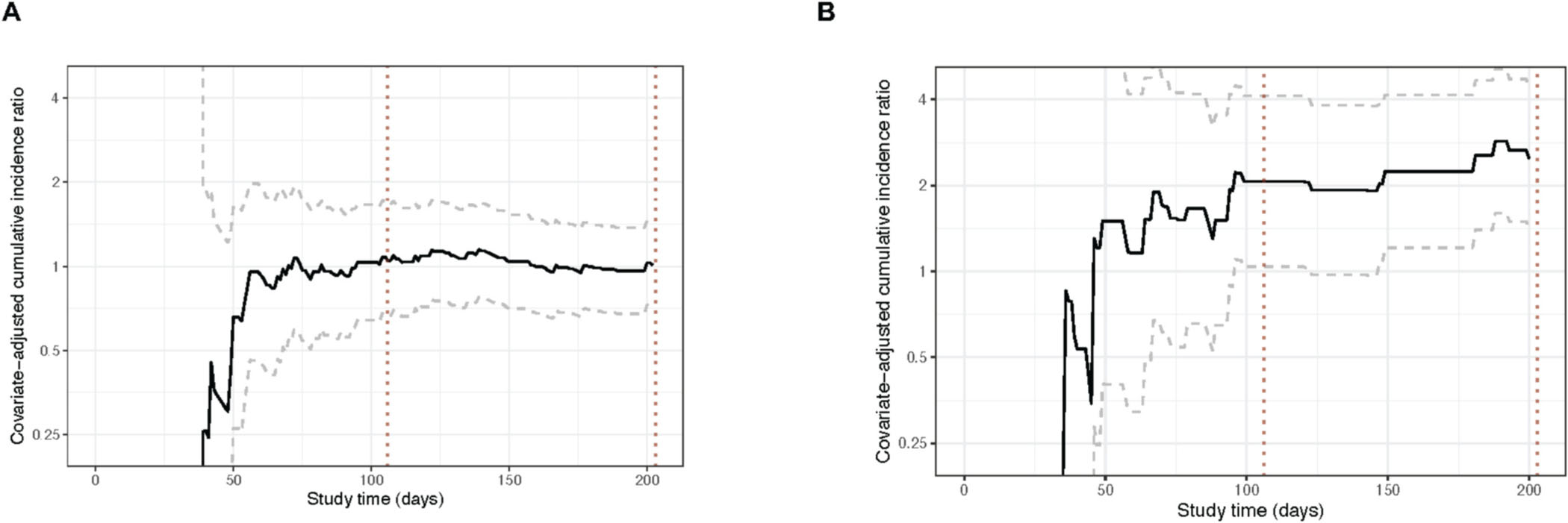
Covariate-adjusted, marginalized cumulative incidence ratio comparing Prototype vs. Omicron-containing vaccines including COVID-19 endpoints starting 7 days post Day 15 visit through 188 days post Day 15 visit for **(A)** Moderna Stage 1 and **(B)** Pfizer/BioNTech Stage 2. Omicron-based vaccines comprise (Beta + Omicron BA.1, Delta + Omicron BA.1, Omicron BA.1, Omicron BA.1 + Prototype) pooled for Moderna and (Beta + Omicron BA.1, Omicron BA.1, Omicron BA.1 + Prototype) pooled for Pfizer/BioNTech. Solid lines are point estimates, dashed lines are 95 % pointwise confidence intervals. The booster-proximal cases (occurring between 7 and 91 days after Day 15 visit) and booster-distal cases (between 92 and 188 days post Day 15 visit) are divided by the red vertical dashed lines. Study time is days since booster.

Fig. 1A shows the covariate-adjusted cumulative incidence curves for recipients of the Moderna monovalent Prototype and Omicron-based mRNA vaccines in Stage 1, while Fig. 2A shows the covariate-adjusted cumulative incidence ratio over time (Prototype vs. Omicron-based). Fig. 1B and Fig. 2B show the corresponding cumulative incidence curves and incidence ratios for the Pfizer/BioNTech monovalent Prototype and Omicron-based mRNA vaccines in Stage 2. Taken together, these show overlap of the 95 % confidence intervals of the incidence curves for the two Moderna groups (cumulative incidence risk ratio [RR] through 188 days post-D15 = 1.04; 95 % CI: 0.73 to 1.48). For the Pfizer/BioNTech vaccines, the cumulative incidence was significantly higher in recipients of the monovalent Prototype vaccine compared to the Omicron-based vaccines (RR = 2.56; 95 % CI: 1.44 to 4.52).

Calendar time-based Cox regression analysis, stratified on a participant’s baseline prior infection status and adjusted for the risk score, was performed separately for Stage 1 and Stage 2 participants to compare incidence of COVID-19 between monovalent Prototype and Omicron-based booster vaccinations. In Stage 1, there was no difference in incidence (hazard ratio [HR] = 1.04 [95 % CI, 0.68–1.58), *p* = 0.86), while in Stage 2, there was a significantly higher incidence in participants who received the monovalent Prototype compared to those receiving an Omicron-based vaccine (HR = 2.95 [95 % CI, 1.52–5.72], *p* = 0.001). The difference in cumulative incidence of COVID-19 between recipients of the Pfizer/BioNTech Prototype versus Omicron-based vaccines remained statistically significant whether restricted to the booster-proximal period (HR = 2.51 [95 % CI, 1.19–5.3], *p* = 0.016) or the booster-distal period (HR = 5.37 [95 % CI, 1.29–22.32], *p* = 0.021).

The difference in protection between Prototype and Omicron-based vaccines observed in Stage 2 was seen regardless of prior infection status, although the difference in incidence in non-naïve participants was not statistically significant likely due to the small number of cases (*n* = 7 cases in 70 individuals) in this subgroup (Supplementary Fig. 2). Furthermore, the difference in COVID-19 incidence between Prototype and Omicron-based vaccine recipients in Stage 2 held even when only cases of COVID-19 due to Omicron BA.4 and BA.5 were included in the analysis (Supplementary Fig. 3).

The cumulative incidence of COVID-19 did not appear to differ substantially between arms that received bivalent Prototype-Omicron, monovalent Omicron, and non-Prototype bivalent Omicron vaccines, either in Stages 1 or 2 (Supplementary Fig. 4), although the number of events in each arm were too small to analyze statistically.

### 3.5. SARS-CoV-2 variant sequencing of breakthrough infections

**Fig. 3.**
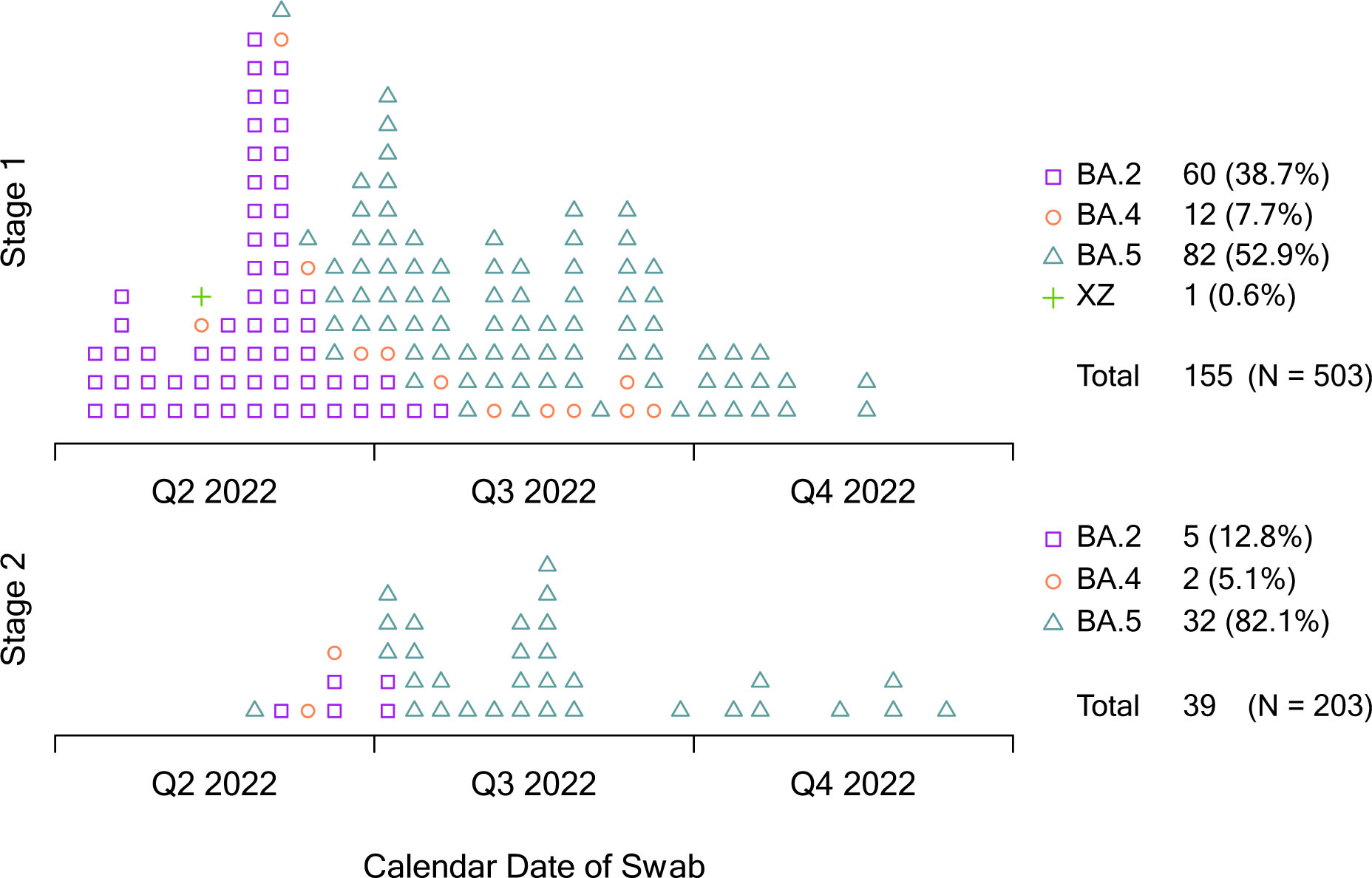
Lineages of the 194 incident COVID-19 breakthrough cases starting 7 days post Day 15 visit through 188 days post Day 15 visit included in the analyses for (A) Moderna Stage 1 and (B) Pfizer/BioNTech Stage 2. The lineages of 48 COVID-19 endpoints were imputed based on the modal circulating lineage on the date of COVID-19 onset according to the GISAID database (matched by date and state or District of Columbia).

Complete viral genomes and lineage assignments were obtained for 146 of the 194 incident COVID-19 breakthrough cases detected from study days 15 through 6 months later in both Stage 1 and 2 participants (Fig. 3). The lineages of the other 48 endpoints were imputed based on the modal circulating lineage on the date of COVID-19 onset according to the GISAID database (matched by date and state or District of Columbia). Likely due to the earlier enrollment of Stage 1, a higher proportion of cases were due to Omicron BA.2 (38.7 %; 60 of 155) in participants who received a single Moderna mRNA booster dose compared to 12.8 % (5 of 39) in Stage 2 participants who received a single Pfizer/BioNTech mRNA booster dose. Conversely, more COVID-19 cases in Pfizer/BioNTech vaccine Stage 2 participants were due to Omicron BA.5 (82.1 %; 32 of 39) compared to Moderna vaccine recipients in Stage 1 (52.9 %; 82 of 155).

## 4. Discussion

Since the beginning of the COVID-19 pandemic, new SARS-CoV-2 variants have continually emerged due to selection pressure from vaccine- and infection-induced immunity. These have led to breakthrough infections that have prompted reformulation of COVID-19 vaccines to target emerging variants of concern. Hypothetically, and similar to the experience with influenza, updated vaccines that are more closely related to currently circulating strains would result in improved vaccine efficacy compared to those targeting more distantly related strains. While logical, strain matching has not, to our knowledge, been based on evidence from randomized trials with clinical endpoints.

When Stages 1 and 2 of the COVAIL study were enrolled (March–May 2022), the COVID-19 vaccines that were authorized for use as booster doses were still based on the Prototype strain. However, by that time, the Omicron strain had already appeared, including subvariant BA.2, rapidly followed by BA.4 and BA.5. The COVAIL trial was conducted primarily to evaluate the immunogenicity and safety of SARS-CoV-2 variant vaccines, although an exploratory endpoint was to identify and evaluate breakthrough infections by sequencing strains for variant spike protein lineage. Here we describe the incidence of symptomatic SARS-CoV-2 infection in study participants who received a second booster dose of either the monovalent Prototype mRNA vaccine or an investigational monovalent or bivalent product that included mRNA encoding for the Spike sequence of Omicron BA.1, produced by either Moderna (Stage 1) or Pfizer/BioNTech (Stage 2). A strength of this study is that each stage randomized participants to receive Prototype or Omicron-based booster doses, such that within each stage the comparative results on cumulative incidence should be valid.

Notably, although no difference in cumulative incidence of COVID-19 cases was observed in those who received the Moderna Prototype mRNA vaccine compared to one of the Omicron-based vaccines in Stage 1, a higher level of protection was seen in Stage 2 in those who received a Pfizer/BioNTech Omicron-based vs. Prototype vaccine. This finding was consistent regardless of prior infection status and whether the analysis was restricted to incident cases of COVID-19 due to Omicron BA.4/5 or included all recorded COVID-19 cases and held after adjusting for baseline infection status and risk score.

Although the differential results between Stage 1 (Moderna) and Stage 2 (Pfizer/BioNTech) are intriguing, there are several limitations of the analysis that may impact their interpretation. First, participants were not randomized between Stage 1 vs. 2, follow-up was staggered to be 1–2 months later for Stage 2, and the study was not designed to make direct comparisons between stages or vaccine products. Second, Stage 1 included a Moderna Delta + Omicron vaccine arm not included in Stage 2 (Pfizer/BioNTech vaccines). Furthermore, asymptomatic infections were not captured, and among the cases of COVID-19 that were included in the analysis there were no reported hospitalizations or deaths. In contrast to the results of this analysis in which there was no difference in vaccine efficacy between Prototype and Omicron-based vaccines in Stage 1, other studies have shown that vaccines that are more closely matched to circulating strains in terms of the spike sequence are more protective against hospitalization and medically-attended COVID-19 [14,15].

While the reason for the observed difference in cumulative incidence between Prototype versus Omicron-based mRNA vaccines in Stage 2 but not Stage 1 is not clear, one compelling possibility is the higher standardized FOI score in Stage 1. A higher force of infection has been suggested to negatively impact vaccine efficacy [16]. Hypothetically, the higher FOI to which Moderna vaccine recipients were exposed in Stage 1 may have overwhelmed any potential relative advantage in efficacy afforded by the Omicron-based vaccines that was antigenically closer to the contemporaneous circulating strains, compared to Prototype. However, the mean unstandardized FOI score, on the scale of time-averaged number of cases per 100,000 persons during an individual’s approximately 6-month follow-up, was only slightly larger for Stage 1 compared to Stage 2: 12.7 cases per 100 K persons per day vs. 11.0 cases per 100 K persons per day, respectively.

Given that neutralizing antibody levels are strongly predictive of protection from SARS-CoV-2 infection and COVID-19 disease [17–20], differences in COVID-19 case incidence between COVAIL trial Stages 1 and 2 may be expected to have been due to variation in the SARS-CoV-2 neutralization responses in recipients of Pfizer/BioNTech compared to Moderna products. However, significant differences in the pattern of pseudovirus neutralization responses post-booster dose were not seen between the Moderna and Pfizer/BioNTech vaccines in COVAIL. Equivalent absolute day 15 mean neutralization levels as well as fold increases in in neutralization from baseline to day 15 against Omicron variant BA.1 compared to D614G were observed for both BioNTech and Moderna Omicron-based vaccines, although these were higher than for the Prototype vaccines in both stages. The similar neutralizing antibody responses in Omicron-based vaccine recipients compared to Prototype in Stages 1 and 2 make it unlikely that they contributed significantly to the observed differences in relative efficacy between these two study groups. Therefore, it does not seem plausible that variability in the humoral immune response could account for the difference in COVID-19 incidence seen between Prototype and Omicron-based vaccines in Stage 2 but not in Stage 1, especially since previous studies have shown that variability in incidence is associated with relatively large differences in neutralization titer [21].

Despite inducing similar absolute antibody and neutralization levels, functional differences in vaccine-induced humoral profiles have been observed between the two currently approved mRNA COVID-19 vaccines. For example, significantly higher levels of Fc*γ* receptor binding antibodies have been seen in Moderna Prototype (mRNA-1273) vaccine recipients compared with Pfizer/BioNTech Prototype (BNT162b2) recipients [22]. In the same study, enhanced antibody-dependent cellular phagocytosis, neutrophil phagocytosis, complement deposition, and NK activation were also seen with mRNA-1273 in comparison to BNT162b2.

Furthermore, differences in cellular immune responses to mRNA-1273 and BNT162b2 have been described. Although both vaccines induce robust CD4+ and CD8+ T cell responses, mRNA-1273 produces higher overall responses compared to BNT162b2, particularly for memory CD4+T cells [23,24]. Given the observation that T cell epitopes are less susceptible to mutations than those targeted by neutralizing antibodies, vaccines that induce stronger T cell responses may be less susceptible to immune escape by new variants [25].

Although the two approved COVID-19 vaccines based on the mRNA platform are similar in composition, they are not identical and vary in factors such as the mRNA dose (30 μg in the Pfizer/BioNTech vaccines vs. 50 μg in the Moderna ones) and lipid nanoparticle composition. Whether the lower dose of mRNA in the Stage 2 vaccines contributed to the lower relative efficacy observed in Prototype recipients compared to Omicron-based vaccine recipients, compared to no difference seen in the corresponding Stage 1 study groups, remains to be determined. Since the only Omicron vaccine insert evaluated was BA.1 (B.1.1.529), it is unknown whether similar results would have been seen with inclusion of mRNA encoding spike of later Omicron variants.

Another factor that may have impacted the relative efficacy of Omicron-based vs. Prototype vaccines in the two stages was the predominant circulating SARS-CoV-2 strain during the periods of observation. Although the staggered enrollment into the two stages was only separated by approximately 2 months, this coincided with the transition from Omicron BA.2 predominance to BA.5. Although BA.2 is more closely related to the Omicron component in both Moderna and Pfizer/BioNTech vaccines tested in COVAIL, in Stage 2, the Prototype vaccine spike sequence was even less well matched to the predominant circulating BA.5 strain compared to Stage 1. Moreover, Stage 1 Omicron-containing vaccine recipients had slightly greater neutralization potency against BA.4/5 than Stage 2 Omicron-containing vaccine recipients.

Additionally, increased transmissibility of the BA.5 Omicron subvariant [26], suggested by the larger BA.5 wave of infections compared to the BA.2 wave, may also have impacted the observed differences in vaccine efficacy between the two COVAIL stages.

Finally, although small, there were significant differences in the study populations between the two stages of COVAIL that may have contributed to the variable efficacy responses that were observed. Although not statistically significant, Stage 1 (Moderna) participants were slightly older than Stage 2 (Pfizer/BioNTech) participants; however, they were significantly less likely to have reported prior infection or tested positive for anti-N antibodies at enrollment and had shorter time since the most recent vaccination or infection. When stratifying the analysis by prior infection status, the advantage of Omicron-matching for Pfizer/BioNTech vaccination was remarkably consistent across all subgroups, whereas the similarity of risk between Moderna Omicron-matched vs. Prototype vaccination was clearest for participants without prior infection but trended toward an Omicron-based vaccine advantage for subgroups with prior evidence of infection. In general, stratified analyses by prior infection status are informative, and a strength of this study is the sufficient sample size to answer the study objective for the Stage 1 infection-naïve, Stage 2 naïve, and Stage 2 non-naïve subgroups, but is limited by insufficient sample size to answer the question for the Stage 1 non-naïve subgroup.

In conclusion, we demonstrated that Omicron-based mRNA SARS-CoV-2 vaccines administered as second booster doses were more protective against COVID-19 relative to Prototype vaccine among those randomized to BNT162b2 but not mRNA-1273. Differences between study stages such as force of infection, study population, antigen dose, and antigen matching may have been contributing factors to this observed difference in clinical efficacy.

## Supplementary Material

1

## Figures and Tables

**Table 1:** COVAIL Study Arms* Included in Analysis of Cumulative COVID-19 Incidence.

Stage	Arm	N	Vaccine Candidate

Stage 1 Moderna mRNA Vaccine	1	97	Prototype 50 μg
	2	111	Beta (B.1.351) 25 μg + Omicron BA.1 (B.1.1.529) 25 μg
	4	100	Delta (B.1.617.2) 25 μg + Omicron BA.1 (B.1.1.529) 25 μg
	5	99	Omicron BA.1 (B.1.1.529) 50 μg
	6	96	Omicron BA.1 (B.1.1.529) 25 μg + Prototype 25 μg
Stage 2 Pfizer-BioNTech BNT Vaccine	7	47	Prototype 30 μg
	8	51	Beta (B.1.351) 15 μg + Omicron BA.1 (B.1.1.529) 15 μg
	9	53	Omicron BA.1 (B.1.1.529) 30 μg
	10	52	Omicron BA.1 (B.1.1.529) 15 μg + Prototype 15 μg

* Arm 3 is not included as it did not include a Prototype or Omicron-based vaccine.

**Table 2:** Baseline Characteristics Including Pseudovirus Neutralization Titers of Stage 1 and 2 Participants Included in the Analysis of Cumulative COVID-19 Incidence.

	Stage 1 Moderna mRNA Vaccines				Stage 2 Pfizer-BioNTech Vaccines				Omicron-based (Stage 1 vs. 2)	Prototype (Stage 1 vs. 2)
				
Variable	Overall (n = 503)	Omicron-based (*n* = 406)	Prototype (*n* = 97)	*P* ^a^	Overall (*n* = 203)	Omicron-based (*n* = 156)	Prototype (*n* = 47)	*P* ^a^	*P* ^b^	*P* ^b^

Age, mean (SD)	51.9 (17.4)	51.5 (17.5)	53.7 (16.5)	0.251	50.3 (16.8)	50.1 (16.5)	51.1 (17.7)	0.725	0.392	0.378
Age ≥ 65 (%)	174 (34.6)	140 (34.5)	34 (35.1)	1	59 (29.1)	45 (28.8)	14 (29.8)	1	0.241	0.660
Non-white (%)	78 (15.5)	60 (14.8)	18 (18.6)	0.443	42 (20.7)	31 (19.9)	11 (23.4)	0.75	0.180	0.647
No prior infection (%)	400 (79.5)	324 (79.8)	76 (78.4)	0.858	133 (65.5)	103 (66.0)	30 (63.8)	0.918	0.001	0.099
Standardized FOI, mean (SD)	0.58 (0.64)	0.58 (0.63)	0.57 (0.69)	0.87	0.00 (0.66)	0.01 (0.68)	−0.03 (0.61)	0.714	<0.001	<0.001
Risk score, mean (SD)	−1.44 (0.38)	−1.43 (0.38)	−1.48 (0.39)	0.263	−1.44 (0.30)	−1.42 (0.30)	−1.49 (0.29)	0.12	0.659	0.833
Pseudovirus Neutralization Titer ID50 [mean (log10-scale) (SD)]										
D614G	3.56 (0.64)	3.57 (0.65)	3.52 (0.62)	0.477	3.63 (0.62)	3.61 (0.63)	3.70 (0.62)	0.387	0.499	0.496
Beta	2.97 (0.74)	2.98 (0.74)	2.92 (0.73)	0.455	3.03 (0.82)	3.00 (0.82)	3.12 (0.81)	0.367	0.485	0.534
Delta	3.26 (0.66)	3.27 (0.66)	3.21 (0.67)	0.484	3.29 (0.68)	3.27 (0.68)	3.38 (0.68)	0.311	0.499	0.493
BA.1	2.49 (0.78)	2.50 (0.78)	2.43 (0.77)	0.404	2.55 (0.88)	2.52 (0.87)	2.65 (0.91)	0.378	0.490	0.511
BA.4/5	2.23 (0.78)	2.24 (0.78)	2.20 (0.77)	0.654	2.27 (0.79)	2.25 (0.80)	2.34 (0.77)	0.496	0.493	0.505
MDW	2.78 (0.70)	2.79 (0.70)	2.73 (0.69)	0.472	2.83 (0.75)	2.81 (0.75)	2.92 (0.75)	0.383	0.492	0.510

Abbreviations: FOI, standardized force of infection score, average of COVID-19 incidence rates (state or DC); ID_50_, serum inhibitory dilution required for 50 % neutralization; MDW, maximal signal diversity weighted average of antibody markers; SD, standard deviation.

a Within-stage comparison between monovalent Protype and Omicron-based mRNA vaccines (Student’s t-test for continuous variables and chi-squared test for categorical variables).

b Comparison between Stage 1 and Stage 2 monovalent Prototype mRNA vaccines or Omicron-based mRNA vaccines (Student’s *t*-test for continuous variables and chi-squared test for categorical variables. Comparison of baseline pseudovirus neutralization titer ID_50_ adjusted for age, sex, prior infection and risk score).

**Table 3:** Neutralization Titers of Stage 1 and 2 Participants^a^ to Omicron BA.1 and Ancestral (D614G) Pseudoviruses.

		Omicron BA.1			D614G			Ratio of geometric mean D15 BA.1 vs. D614G	Ratio of geometric mean fold rise to BA.1 vs. D614G
			
Stage	Vaccine	Mean Baseline (log_10_-scale)	Mean D15 (log_10_-scale)	Geometric Mean Fold Rise	Mean Baseline (log_10_-scale)	Mean D15 (log_10_-scale)	Geometric Mean Fold Rise		

1	Omicron-Containing	2.51	3.70	15.50 (13.50, 17.90)	3.58	4.35	5.86 (5.20, 6.59)	0.225 (0.207, 0.244)	2.65 (2.38, 2.95)
1	Prototype	2.44	3.42	9.68 (7.53, 12.40)	3.53	4.32	6.18 (5.16, 7.41)	0.127 (0.106, 0.152)	1.57 (1.33, 1.84)
2	Omicron-Containing	2.54	3.65	12.80 (10.20, 16.10)	3.63	4.28	4.48 (3.81, 5.26)	0.233 (0.211, 0.257)	2.87 (2.47, 3.33)
2	Prototype	2.65	3.42	5.85 (4.08, 8.38)	3.70	4.33	4.22 (3.24, 5.51)	0.123 (0.097, 0.155)	1.38 (1.10, 1.74)

a Participants who experienced early infections (prior to 7 days post-D15) were excluded.

## Data Availability

Data will be made available on request.

## Appendix A. Supplementary data

Supplementary data to this article can be found online at https://doi.org/10.1016/j.vaccine.2025.127718.
