# Supplementary material for: Effect of Omicron BA.1-based compared to prototype booster mRNA vaccination on incidence of COVID-19 in the COVAIL trial
Source: Vaccine. Author manuscript; Available in PMC 2026 Jun 30. (PMC13316942; doi:10.1016/j.vaccine.2025.127718)
Supplement: 1 [file NIHMS2182316-supplement-1.docx]

**COVAIL Manuscript Study Group**

**George Washington University, Washington D.C.**

David J. Diemert, MD; Elissa Malkin, DO; Jeffrey M. Bethony, PhD; Aimee Desrosiers, PA-C; Marc Siegel, MD

**University of Rochester VTEU, Rochester, NY**

Angela R. Branche, MD; Ann R. Falsey, MD; Edward Walsh, MD; Patrick Kingsley, BS; Michael Peasley, BS

**Emory University Hope Clinic, Decatur, GA**

Nadine G. Rouphael, MD; Cecilia Losada, MD; Daniel S. Graciaa, MD; Hady Samaha, MD; Paulina A Rebolledo, MD; Zanthia Wiley, MD.

**Brigham and Women’s Hospital, Harvard Medical School, Boston, MA**

Lindsey R. Baden, MD; Amy C. Sherman, MD; Stephen R. Walsh, MD; Alexandra Tong, BS; Rebecca Rooks, BS

**Saint Louis University, St. Louis, MO**

Sharon E. Frey, MD; Getahun Abate, MD, PhD; Zacharoula Oikonomopoulou, MD; Daniel F. Hoft, MD, PhD; Irene Graham, MD

**Departments of Molecular Virology and Microbiology and Medicine, Baylor College of Medicine, Houston, TX**

Jennifer A. Whitaker, MD; Hana M. El Sahly, MD; Wendy A. Keitel, MD; C. Mary Healy, MD; Robert L. Atmar, MD

**Department of Medicine, Division of Infectious Diseases and Global Public Health, University of California San Diego, La Jolla, CA**

Susan J. Little, MD; Thomas C.S. Martin, MD; Nicole Carter, MPH; Steven Hendrickx, RN

**Center for Childhood Infections and Vaccines (CCIV) of Children’s Healthcare of Atlanta and Emory University Department of Pediatrics, Atlanta, GA**

Evan J. Anderson, MD; Christina A. Rostad, MD; Satoshi Kamidani, MD; Etza Peters, RN

**Duke Human Vaccine Institute, Duke University School of Medicine, Durham, NC**

Emmanuel B. Walter, MD, MPH; Michael J. Smith, MD, MSCE; M. Anthony Moody, MD; Kenneth E. Schmader, MD

**University of Illinois at Chicago-Project WISH, Chicago, IL**

Richard M. Novak, MD; Benjamin G. Ladner, MD; Andrea Wendrow, RPh; Jessica Herrick, MD

**University of Texas Medical Branch, League City, TX**

Richard Rupp, MD; Laura Porterfield, MD

**Kaiser Permanente Washington Health Research Institute, Seattle, WA**

Lisa A. Jackson, MD, MPH; Maya Dunstan, MS, RN; Rebecca Lau, PharmD; Barbara Carste, MPH

**Department of Medicine, University of Washington, Seattle, WA**

Tara M. Babu, MD, MSCI; Anna Wald, MD, MPH; Taylor Krause, BA; Kirsten Hauge, MPH

**NYU VTEU Manhattan Research Clinic at NYU Grossman School of Medicine, New York, NY**

Angelica C. Kottkamp, MD; Mark J. Mulligan, MD; Tamia Davis, NP; Celia Engelson, NP; Vijaya Soma, MD

**Zuckerberg San Francisco General, University of California San Francisco, San Francisco, CA**

Anne F. Luetkemeyer, MD; Chloe Harris, BA; Azquena Munoz Lopez, BS

**Morehouse School of Medicine, Atlanta, GA**

Lilly C. Immergluck, MD; Erica Johnson, PhD; Austin Chan, MD

**Washington University School of Medicine, St. Louis, MO**

Rachel M. Presti, MD, PhD; Jane A. O’Halloran, MD, PhD; Ryley M. Thompson

**NYU VTEU Long Island Research Clinic at NYU Long Island School of Medicine, Mineola, NY**

Martín Bäcker, MD; Andrew B. Fleming, MD; Asif Noor, MD

**University of Iowa College of Medicine, Iowa City, IA**

Patricia L. Winokur, MD; Jeffery Meier, MD; Jack Stapleton, MD

**Howard University College of Medicine, Howard University Hospital, Washington D.C.**

Siham M. Mahgoub, MD; Celia Maxwell, MD; Sarah Shami, PharmD

**University of Alabama at Birmingham, Birmingham, AL**

Paul A. Goepfert, MD

**Tulane University School of Medicine, New Orleans, LA**

Dahlene N. Fusco, MD; Arnaud C. Drouin, MD; Florice K. Numbi, MD

**University of Maryland, Baltimore, MD**

Kirsten E. Lyke, MD

**IDCRC Principal Investigators**

David S. Stephens, MD; Kathleen M. Neuzil, MD

**IDCRC Leadership Operations Center**

Monica M. Farley, MD; Jeanne Marrazzo, MD; Sidnee Paschal Young

**IDCRC Clinical Operations Unit**

Jeffery Lennox, MD; Robert L. Atmar, MD; Linda McNeil FHI360

**IDCRC Statistical and Data Science Unit**

Elizabeth Brown, PhD

**IDCRC Laboratory Operations Unit – Fred Hutchinson Cancer Center, Seattle, WA**

Christine M. Posavad, PhD; Megan A. Meagher, BS; Julie McElrath, MD; Mike Gale, PhD

**FHI360, Durham, NC**

Kuleni Abebe, MSc

**The Emmes Company, LLC, Rockville, MD**

Mat Makowski, PhD; Heather Hill, MS; Jim Albert, MS; Holly Baughman; Lisa McQuarrie, MS; Kalyani Telu, MS; Jinjian Mu, PhD; Lisa J. McQuarrie^,^ MSc

**Clinical Monitoring Research Program Directorate, Frederick National Laboratory for Cancer Research, Frederick, MD**

Teri C. Lewis, BS; Lisa A. Giebeig, MS; Theresa M. Engel, MFS; Caleb J. Griffith, MPH; Wendi L. McDonald, BSN; Alissa E. Burkey, MS; Lisa B. Hoopengardner, MS; Jessica E. Linton, MS; Nikki L. Gettinger, MPH

**Department of Surgery and Duke Human Vaccine Institute, Duke University School of Medicine, Durham, NC**

David C. Montefiori, PhD; Amanda Eaton, MBA

**Smith’s Laboratory, Cambridge, UK**

Derek J. Smith, PhD; Antonia Netzl; Samuel H. Wilks, PhD; Sina Türeli, PhD

**Division of Microbiology and Infectious Diseases, National Institute of Allergy and Infectious Diseases, National Institutes of Health, Bethesda, MD**

Mamodikoe Makhene, MD; Mohamed Elsafy, MD; Rhonda Pikaart-Tautges, BS; Janice Arega, MS; Binh Hoang, RPh; Dan Curtin; Hyung Koo, BSN; Elisa Sindall, BSN; Sonja Crandon, BSN; Marciela M. DeGrace, PhD; Diane J. Post, PhD; Seema U. Nayak, MD; Paul C. Roberts, PhD; John H. Beigel, MD

**Department of Microbiology, Icahn School of Medicine at Mount Sinai, New York, NY**

Viviana Simon, MD, PhD; Giulio Kleiner, PhD; Komal Srivastava, MS

**Department of Genetics and Genomic Sciences, Icahn School of Medicine at Mount Sinai, New York, NY**

Harm van Bakel, PhD; Zain Khalil, MS; Ana Silvia Gonzalez-Reiche, PhD

**COVAIL Manuscript Study Team Members**

**Emory University Hope Clinic, Decatur, GA**

Nadine G. Rouphael, MD; Cecilia Losada, MD; Daniel S. Graciaa, MD; Hady Samaha, MD; Cassie Grimsley Ackerley, MD; Kristen E. Unterberger, PA; Amy Anderson, BSN; Mary Atha, ACNP; Kareem Bechnak, BSN; Sarah Bechnak, BSN; Mary Bower, BSN; Laura Clegg, RN; Matthew Collins, MD, PhD; Francine Dyer, RN; Srilatha Edupuganti, MD; Rebecca Fineman, BS; Tigisty Girmay, MSN; Rebecca Gonzalez, PharmD; Natalie Gray, BS; Evan Gutter, MPH; Lisa Harewood; Chris Huerta, MSc; Brandi Johnson, BS; Lauren Johnson, MPH; Colleen Kelley, MD; Alexandra Koumanelis, BA; Deborah Laryea, BSN; Hollie Macenczak, BSN; Nour Makkaoui, MD; Michele McCullough, MPH; Tuong-Vy Ngo, PharmD; Eileen Osinski, BS; Julia Paine, BS; Bernadine Panganiban, BS; Rose Pope, RN; Paulina Rebolledo, MD; Susan Rogers, RPh; Erin Scherer, PhD; Veronica Smith, NP-C; Andre Stringer, BS; Jessica Traenkner, PA; Dongli Wang, BS; Alahna Watson, BA; Stacey Wheeler, RN; Jean Winter; Jianguo Xu, PhD

**Brigham and Women’s Hospital, Harvard Medical School, Boston, MA**

Lindsey R. Baden, MD; Amy C. Sherman, MD; Stephen R. Walsh, MD; Alexandra Tong, BS; Rebecca Rooks, BS; Jane A. Kleinjan, NP; Jon A. Gothing, NP; Andres A. Avila Paz, BA; Muneerah M. Aleissa, PharmD, MPH; Bethany Evans, BA; August Heithoff, BS; Natalie E. Izaguirre, MS; Hannah Jin, MPH; Urwah Kanwal, BS; Austin Kim, BS; Julia E. Klopfer, BS; Christina Montesano, BS; John Almeida, BA; Emily S. Koleske, BS; Hannah Levine, BS; Nicholas P. Morreale, BS; Omolola Ometoruwa, BS; Jun Bai Park Chang, BS; Anna F. Piermattei, BA; Djenane M. Pierre, BS; Megan Powell, BA; Kevin Zinchuk, PharmD; Stephanie Pickford, PharmD; Charles M. Kelly III, PharmD; Xiaofang Li, PhD; John Kupelian, BS ; Kimberly Dufresne, BS; Xiaoguang Fan, MD, PhD; Xi Zhang, PhD; Esther Arbona-Haddad, MD; Jose Humberto Licona, MD

**Center for Childhood Infections and Vaccines (CCIV) of Children’s Healthcare of Atlanta and Emory University Department of Pediatrics, Atlanta, GA**

Evan J. Anderson, MD; Christina A. Rostad, MD; Satoshi Kamidani, MD; Etza Peters, RN; Larry Anderson, MD; Julia Bartol; Leisa Bower, RN; Natsuko Campbell, RN; Lisa Harewood; Hui-Mien Hsiao; Laila Hussaini, MPH; Inara Jooma; Gidget Kettle, RN; Marcia Lewis, RN; Wensheng Li; Cindy Lubbers, RN; Lisa Macoy, RN; Molly Morrison, Heather Nurse, RN; Anna Siaw-Anim; Kathleen Stephens, RN; Madeline Taylor; Ashley Tippett, MPH; Lauren Nolan, PA

**Zuckerberg San Francisco General, University of California San Francisco, San Francisco, CA**

Anne F. Luetkemeyer, MD; Chloe Harris, BA; Azquena Munoz Lopez, BS; Daniel Berrner; Dennis Dentoni-Lasofsky, MSN; John Dwyer, RN; Suzanne Hendler, BSN; Elvira Gomez, MPH; WeyLing Phuah, PharmD; Jaime Velasco, BA; Veronica Viar, MS

**George Washington University, Washington D.C.**

David J. Diemert, MD; Elissa Malkin, DO; Jeffrey M. Bethony, PhD; Aimee Desrosiers, PA-C; Marc Siegel, MD; Nikita Schroll-McLaughlin, MS; Jonathan Manning, BA; Jane Ryu, MS; Hanna-Grace Rabanes, MPH; Khadija Khan, MPH; Laura Vasquez, MPH; Caroline Thoreson, PA-C; Larissa Scholte, PhD; Rafaela Thur, DVM; Peyton St. John, BS; Dorinne Mettle-Amuah, PharmD

**University of Iowa College of Medicine, Iowa City, IA**

Patricia L. Winokur, MD; Jeffery Meier, MD; Jack Stapleton, MD; Laura Stulken, PA; Theresa Hegmann, PA; Deb Pfab, RN; Elizabeth Morgan, RN; Susan Herman, RN; Angel Peguero, CMA; Michelle Rodenburg; Alfred J. Carr; Delilah Johnson

**Washington University School of Medicine, St. Louis, MO**

Rachel M. Presti, MD, PhD; Jane A. O’Halloran, MD, PhD; Michael Klebert, RN, PhD; Ryley M. Thompson; Alem Haile; Kim Gray, NP; Chapelle Ayres; Delaney Carani, RN; Michael Royal; John Tran; Laura Blair; Anita Afghanzada; Natalie Schodl

**NYU VTEU Manhattan Research Clinic at NYU Grossman School of Medicine, New York, NY**

Angelica C. Kottkamp, MD; Tamia Davis, NP; Celia Engelson, NP; Vijaya Soma, MD; Abdulwahab Abdulai; Ashanay Allen; Natella Aronova, NP; Philip Aziz, PharmD; Emily Beato; Samuel Bliss, PharmD; Jacqueline Callahan, RN; Ellie Carmody, MD; Amanda Dontino, BS; Aimee Edwin, RN; Shelby Goins; Sarah Haiken; Ramin Herati, MD; Abdonnie Holder; Janice Hong; Trishala Karmacharya; Manpreet Kaur, PharmD; Hye-Youn Kim; Alexander McMeeking, MD; Mark Mulligan, MD; Wai Ng; Edward Nirenberg; Irma Noriega, NP; Samuel Nweke; Lalitha Parameswaran, MD; Levonne Phillip, MPH; Stephanie Rettig, MPH; Marie Samanovic-Golden, PhD; Madalyn Saporito; Pamela Suman; Meron Tasissa; Michael Tuen; Julia Wagner, MPH; James Wilson; Doris Wong, PharmD; Grace Yip, BS; Samantha Yip, RN; Heekoung Youn, RN; Lisa Zhao

**University of Rochester VTEU, Rochester, NY**

Angela R. Branche, MD; Ann R. Falsey, MD; Edward E. Walsh, MD; Patrick Kingsley, BS; Arthur Zemanek, BSN, MS; Katherine Elena, BSN; Spencer Obrecht, BSN; Ian Shannon, BSN; Amy Kaychalo, BS, MS; Erin Nowicki; Sharon Moorehead; Kari Steinmetz, BA; Doreen Francis, RN; Tanya Smith, BS; William Hamilton, BS; Jeanne Holden-Wiltse, MPH, MBA; Christopher Lane, MS; Michael Peasley, BS; Samuel Diehl, BS; Kyle Richards, PharmD; Stephen Bean, PharmD; Nicole Dornbush, PharmD; Carol Cole, PharmD

**Saint Louis University, St. Louis, MO**

Sharon E. Frey, MD; Getahun Abate, MD, PhD; Zacharoula Oikonomopoulou, MD; Daniel F. Hoft, PhD, MD; Irene Graham, MD; Azra Blazevic, DVM, MPH; Tamara Blevins, MS; Kathleen Chirco, BSN; Sabrina M. DiPiazza, BSN, MA; Stanley Doublin; Heather Hoertel Douds, MSNS, BSN; Carol G. Duane, PhD, RN; Eric Eggemeyer, BA; Linda M. Eggemeyer-Sharpe, BSN; Lauren Nicole Foreman, BSN; Sarah Louise George, MD; Geoffrey J. Gorse, MD; Michelle Harris, PharmD; Helay Hassas, PharmD; Rong Hou, MD; Ryan Clark Kerr, BSN; Kate Elizabeth Liefer, BSN; Melissa J. Loyet, RN; Lainey Mejia-Jauregui, BS; Keith Meyer, BS; Tracy Renee Montauk, BSN; Karla J. Mosby, RN; Amanda Nethington, BS; Huan Ning, MD; Nicole Purcell; Joan M. Siegner, BSN, MA; Janice M. Tennant, BSN, MPH; Mei Xia, PhD; Kiana Wilder, BA; Yinyi Yu, BS; Cassandra Nicole Zehenny, BSN

**University of Texas Medical Branch, League City, TX**

Richard Rupp, MD; Laura Porterfield, MD; Amber Stanford, PA-C; Robert Cox, RN; Kristin Pollock, RN; Diane Barrett, MS; Gerrianne Casey, RN; Amy McMahan, LVN; Cori Burkett, PA-C; Essie Cox

**NYU VTEU Long Island Research Clinic at NYU Long Island School of Medicine, Mineola, NY**

Martín Bäcker, MD; Sarah J. Pastolero, RN; Kimberly Byrnes, RN; Andrew B. Fleming, MD; Asif Noor, MD; Sigridh A. Muñoz-Gómez, MD; Steven E. Carsons, MD; Sajumon K. Joseph, FNP; Sophie Danziger; Monica Benitez; Maung Aung; Louis Ragolia, PhD; Alicia Vasile, RPh; April Correll, RPh; Christopher Hall; Thomas Palaia; Miloni H Thakker, MD; Lavern Harvey; Lisa Zhao; Diana Badillo, MD.

**University of Illinois at Chicago-Project WISH, Chicago, IL**

Richard M. Novak, MD; Benjamin G. Ladner, MD; Andrea Wendrow, RPh; Jesica Herrick, MD; Alfredo J. Mena Lora, MD; Scott A. Borgetti, MD; Diana L Bahena, APRN; Regina Harden, BA; Renyce Powell;  David C. M. Chan, PharmD; Rebeca F. Gasari, PharmD; Michael Pacini, PharmD; Margarita M. Villarreal, CPhT; Rodrigo Reyes, ADN; Samuel M. Rene, MPH; Shannon M Whitted, BSN; Habiba Sultana, MBBS; Nanu Kunwar, BS; Tasmin Sultana, MBBS; Md R. Amin, PhD;  Mahmood Ghassemi, PhD,  Liam Morrissy, BS; Nia O'Neal, BS; Chasity Serrano, BS; Charlie Peterson, BA

**Duke Human Vaccine Institute, Duke University School of Medicine, Durham, NC**

Emmanuel B. Walter MD, MPH; Michael J. Smith MD, MSCE; M. Anthony Moody, MD; Kenneth E. Schmader, MD; Susan Doyle; Lynn S Harrington BSN; Lori Hendrickson BSN; Amy O’Berry MSN; Sherry Huber BSN; Janet Wootton RN, RSCN; Kelly Clark BA; Lani Banez; Stephanie Smith BA; Byron Hauser BS; Ally Odom BA; Emily Randolph BA; Krystina Yoder BA; Kathlene Chmielewski; Luis Ballon BA; Aubree Latorre; Breana Montgomery; Antony Tritz MS; Thad Gurley, MS; Margaret Pendzich

**Kaiser Permanente Washington Health Research Institute, Seattle, WA**

Lisa A. Jackson, MD, MPH; Maya Dunstan, MS, RN; Rebecca Lau, PharmD; Barbara Carste, MPH; Wesley A. Andersen, RPh, MHA, MA; Lee Barr, RN; Cassandra Bryant, BS; Joe Choe, BS; Lynn Gross, PA-C; Erika Kiniry, MPH; Bonnie Y Lam, PharmD; De Vona Lang; Stella Lee, BA; Paula J Lins, PA-C, MPH; Amy Mohelnitzky, PA-C; Marilyn Nguyen, BS; Matthew Nguyen, MPH; Melissa Resendiz Rivas, BA; Melissa Boothe Scheer, PA-C; Janice Suyehira, MD; Stacie Wellwood, LPN; Maryann K Woodford, PA-C

**Department of Medicine, Division of Infectious Diseases and Global Public Health, University of California San Diego, La Jolla, CA**

Susan J. Little, MD; Thomas C.S. Martin, MD; Nicole Carter, MPH; Steven Hendrickx, RN; Ajay Bharti, MD; Alyssa Phillips; Aurora Verduzco Gonzalez, NP; Cheryl Dullano; Chris Houston; Dawn Rosenblum, RN; DeeDee Pacheco; DeLys Brooks; Fang Wan; Helene Le, CPhiT; JC Alcantar; Jill Blumenthal, MD; Joseph Lencioni, MABMH; Kory Hess; Letty Muttera, PharmD; Marlene Arredondo; Megan Smyth; Megan Taylor; Melinda Stafford, PharmD; Michelle Orsburn, MD; Michelle Truong; Niamh Higgins, PharmD, MSc, AAHIVP; Nimish Patel, PharmD, PhD, AAHIVP; Rebecca Gonzalez; Vivian Maldonado

**Morehouse School of Medicine, Atlanta, GA**

Lilly C. Immergluck, MD, MS; Erica Johnson, PhD; Austin Chan, MD; Fatima Ali, MPH; Sonja Jackson; Noor Mohamed, PharmD; LaKesha Tables, MD, MPH; Norberto Fas, MD; Kay Woodson, PharmD; Saadia Khizer, MD; Jacquelyn Ali, MSA; Abdullah Warsama; Eric Gaines; Sierra Jordan Thompson; Cristina Wilson; Trisha Parker, MPH; Xiting Lin; LaTeshia Thomas Seaton, APRN; Derrick Wilson

**Howard University College of Medicine, Howard University Hospital, Washington D.C.**

Siham M. Mahgoub, MD; Celia Maxwell, MD; Sarah Shami, PharmD; Edward Bauer, BS; Yuanxiu Chen, MD, PhD; Megan Ware-Pressley, MHA; Debra Ordor, RN; Linda Fletcher, RN; Emmanuel Baidoo, BS; David Jaspan, RPh, MBA; Adetokunbo Adedokun, PharmD, MPH, BCPS; Michelle Strobeck, BS; Michael A. Riga; Ashley Karen Bautista, BS

**Departments of Molecular Virology and Microbiology and Medicine, Baylor College of Medicine, Houston, TX**

Jennifer A. Whitaker, MD; Hana M. El Sahly, MD; Wendy A. Keitel, MD; C. Mary Healy, MD; Robert L. Atmar, MD; Pedro A. Piedra, MD; Jesus Banay; Kathy Bosworth; Janet Brown, RPh; Kayla Burrell; Jeremy Castro; Tykel Eddy; Marcena Eubanks; Cathy Faw, RPh; Rachel Froebe; Alix Halter, RN; Janey John, MSN, APRN, FNP-C; Chanei Henry, AAS; Vanessa Martinez; Carol Mundell, RN; Brandie Phillips, RN; Alicia Prevost-Barthe, RN; Connie Rangel, RN; Yolanda Rayford, MS; Yvette Rugeley; Maria Shlyapobersky; Tina Sierra; Elizabeth Silguero; Lisreina Toro; Dawn Turner, RN; Chianti Wade-Bowers, RN; Jessica Woods, RN; Robert L. Atmar, MD

**Departments of Medicine, Epidemiology, and Laboratory Medicine & Pathology, University of Washington, Vaccines and Infectious Diseases Division, Fred Hutchinson Cancer Center, Seattle, WA**

Tara M. Babu, MD, MSCI; Anna Wald, MD, MPH; Taylor Krause, BA; Kirsten Hauge, MPH; Jina Taub, ARNP; Dana Varon, ARNP; Britt Murphy, ARNP; Morissa Pertik, PA-C; T. Nui Pholsena, ARNP; Alyssa Braun, BS; ; Jessica Heimonen, MPH; Amy Link, BS; Lindsey McClellan, BS; Jessica Moreno, BS; Chloe Wilkens, BS; Matt Seymour, MPH; Lawrence Hemingway, BS; Jean Mernaugh, BS; Chris McClurkan, BS; Kerry Laing, PhD; Meredith Potochnic, PharmD; Joong Kim, PharmD; Bao-Chao Vo, PhT

**University of Alabama at Birmingham, Birmingham, AL**

Paul A. Goepfert, MD; Jenna Weber, RN; Savannah Spaulding, RN; Heather Logan, CRNP; Faye Heard; Foreamben Patel; Michelle Chambers

**Tulane University School of Medicine, New Orleans, LA**

Dahlene N. Fusco, MD; Arnaud C. Drouin, MD; Florice K. Numbi, MD; Hamada F. Rady, PhD; Crystal A. Ward, MSN; Quinn M. Powers, MS; William E. Casey, BS; Brian P. Logarbo, MD; Shae P. Williams, BS; Emily Callegari, MSN

**IDCRC Principal Investigators**

David S. Stephens, MD; Kathleen M. Neuzil, MD

**IDCRC Leadership Operations Center**

Monica M. Farley, MD; Jeanne Marrazzo, MD; Sidnee Paschal Young

**IDCRC Clinical Operations Unit**

Jeffery Lennox, MD; Robert L. Atmar, MD; Linda McNeil FHI360

**IDCRC Laboratory Operations Unit – Fred Hutchinson Cancer Center and University of Washington, Seattle, WA**

Christine M. Posavad, PhD; Megan A. Meagher, BS; Michael Stirewalt, MBA; John Hural, PhD; Weston Lawler, BA; Lexi Tanser, MA; Julie McElrath, MD, PhD; Mike Gale, PhD

**IDCRC Statistical and Data Science Unit**

Elizabeth Brown, PhD

**University of Maryland, Baltimore, MD**

Kirsten E. Lyke, MD

**FHI360, Durham, NC**

Kuleni Abebe, MSc

**The Emmes Company, LLC, Rockville, MD**

Mat Makowski, PhD; Heather Hill, MS; Jim Albert, MS; Holly Baughman; Lisa McQuarrie, MS; Kalyani Telu, MS; Jinjian Mu, PhD; Lisa J. McQuarrie, MSc

**Clinical Monitoring Research Program Directorate, Frederick National Laboratory for Cancer Research, Frederick, MD**

Teri C. Lewis, BS; Lisa A. Giebeig, MS; Theresa M. Engel, MFS.; Caleb J. Griffith, MPH; Wendi L. McDonald, BSN; Alissa E. Burkey, MS; Lisa B. Hoopengardner, MS; Jessica E. Linton, MS; Nikki L. Gettinger, MPH; Aroussiak Bowen; Beth R. Baseler, MS; Vanessa S. Eccard-Koons, MS; Charles W. R. Hofsommer, JD; Thomas C. Sova, JD; Gary A. Krauss

**Department of Surgery and Duke Human Vaccine Institute, Duke University School of Medicine, Durham, NC**

David C Montefiori, PhD; Amanda Eaton, MBA; Francesca Suman, MS.

**Smith’s Laboratory, Cambridge, UK**

Derek J Smith, PhD; Antonia Netzl; Samuel H Wilks, PhD; Sina Türeli, PhD; Ana Mosterín Höpping, PhD; Samuel Turner; Sarah James, MD; Poppy Roth

**Division of Microbiology and Infectious Diseases, National Institute of Allergy and Infectious Diseases, National Institutes of Health, Bethesda, MD**

Marina Lee, PhD; Mamodikoe Makhene, MD; Mohamed Elsafy, MD; Rhonda Pikaart-Tautges, BS; Janice Arega, MS: Binh Hoang, RPh; Dan Curtin; Hyung Koo, BSN; Elisa Sindall, BSN; Aya Nakamura, RN, MS; Audria Crowder, BS; Guinevere Chun, RN, BSN, MSHS; Frank Kenny, PhD MPH; Seemi Patel, RHP, PharmD; Sonia Gales, MS; Ahsen Khan, JD; Walla Dempsey, PhD; Robert Jurao- RN, BSN; Sonja Crandon, BSN; Seema U. Nayak, MD; Marciela M DeGrace, PhD; Diane J Post, PhD; Paul C Roberts, PhD; John H Beigel, MD; SAVE Program

**Department of Microbiology, Icahn School of Medicine at Mount Sinai, New York, NY**

Viviana Simon, MD, PhD; Giulio Kleiner, PhD; Komal Srivastava, MS; Christian Cognigni, BS; Aria Rooker; Angela Amoako; Dylan Fitzgerald

**Department of Genetics and Genomic Sciences, Icahn School of Medicine at Mount Sinai, New York, NY**

Harm van Bakel, PhD; Zain Khalil, MS; Ana Silvia Gonzalez-Reiche, PhD; Adriana van de Guchte, MS

**Supplementary Table 1. COVID-19 Vaccines Previously Received by Study Participants.**

| **Vaccine**  *(Primary series, 1^st^ boost)* | **Stage 1**  **Moderna mRNA Vaccines**  **N=503**  n (%) | **Stage 2**  **Pfizer-BioNTech Vaccines**  **N=203**  n (%) |
| --- | --- | --- |
| Moderna, Moderna | 165 (32.8%) | 62 (30.5%) |
| Pfizer/BNT, Pfizer/BNT | 260 (51.7%) | 116 (57.1%) |
| Moderna, Pfizer/BNT | 22 (4.4%) | 11 (5.4%) |
| Pfizer/BNT, Moderna | 33 (6.6%) | 12 (5.9%) |
| J&J, J&J | 3 (0.6%) | 0 (0%) |
| J&J, Moderna | 15 (3.0%) | 2 (1.0%) |
| J&J, Pfizer/BNT | 5 (1.0%) | 0 (0%) |

**Supplementary Table 2. Day 15 Neutralization Titers (log_10_-scale) of Stage 1 and 2 Participants to Omicron BA.4/5 Pseudoviruses.** *P*-values comparing Stage 1 and Stage 2 are adjusted for baseline infection-naïve status, age, sex, and risk score.

| **Vaccine** | **Stage 1** | **Stage 2** | ***P*-value** |
| --- | --- | --- | --- |
| Omicron-Based | 3.28 | 3.22 | 0.51 |
| Prototype | 3.06 | 3.03 | 0.50 |

**Supplementary Table 3. COVID-19 Breakthrough Infections in Stage 1 and Stage 2, by Observation Period.**

| **COVID-19 Cases** | **Stage 1**  **Moderna mRNA Vaccines**  n | | **Stage 2**  **Pfizer-BioNTech Vaccines**  n | |
| --- | --- | --- | --- | --- |
|  | Omicron-Containing  N=406 | Prototype  N=97 | Omicron-Containing  N=156 | Prototype  N=47 |
| Total | 127 | 28 | 23 | 16 |
| Early | 11 | 1 | 2 | 0 |
| Booster-Proximal | 70 | 18 | 17 | 12 |
| Booster-Distal | 46 | 9 | 4 | 4 |

**Supplementary Figure 1.** Longitudinal Geometric Mean Neutralization Titers of Stage 1 (Panel A) and Stage 2 (Panel B) Participants to Ancestral (D614G) and Omicron BA.1 Pseudoviruses. Results are plotted only for participants who did not experience incident COVID-19 through 188 days post-D15.

**Supplementary Figure 2.** Forest plot of hazard ratios and 95% confidence intervals for the effect of Omicron-based vaccination *vs*. Prototype vaccination on the incidence of COVID-19 breakthrough infection for both Stages 1 and 2.

**Supplementary Figure 3.** Forest plot of hazard ratios and 95% confidence intervals for the effect of Omicron-based vaccination *vs*. Prototype vaccination on the incidence of COVID-19 breakthrough infection of Omicron BA.4 and BA.5 for both Stages 1 and 2.

**Supplementary Figure 4.** Covariate-adjusted, marginalized cumulative incidence of COVID-19 curves through 188 days post Day 15 visit including COVID-19 endpoints starting 7 days post Day 15 visit for (A) Moderna Stage 1 and (B) Pfizer/BioNTech Stage 2.

A.

B.

**Supplementary Methods**

*Viral Sequencing Methodology*

RNA was extracted from nasopharyngeal swabs resuspended in PBS or VTM solution using the Chemagic™ Viral DNA/RNA 300 Kit H96 (PerkinElmer, cat. CMG-1033-S) on a Chemagic™ 360 instrument (PerkinElmer) per the manufacturer’s protocol. SARS-CoV-2 levels were quantified with the N1 primers/probe set from the CDC/NCIRD/DVD 2019-nCoV real-time RT-PCR Panel (IDT, 2019-nCoV RUO Kit, cat. 10006713). Samples with cycle threshold (Ct) values ≤32 were selected for viral genome amplification and sequencing. cDNA synthesis and whole-genome amplification were performed using two custom primer panels targeting 1.5 and 2 kb regions across the SARS-CoV-2 genome, as previously described [1]. Paired-end (2x150bp) Nextera XT libraries (Illumina, cat. FC-131-1096) were prepared from amplicons and sequenced on a MiSeq instrument. The SARS-CoV-2 genomes were then assembled using the Virus Reference-based Assembly Pipeline and IDentification (vRAPID) package [2]. Finally, complete (>95%) genomes were mapped to major lineages (BA.1, BA.4, BA.5 and XZ) based on Nextclade CLI (v2.13.0 & v2.14.0) partially Aliased assignments [3] and pangolin (v4.1.3 & v4.3) [4].

***Supplementary References:***

1. Gonzalez-Reiche AS, Alshammary H, Schaefer S, et al. Sequential intrahost evolution and onward transmission of SARS-CoV-2 variants. Nat Commun **2023**; 14:3235.

2. Khalil Z, Gonzalez-Reiche AS, Obla A, van Bakel H. vRAPID: Virus Reference-based Assembly Pipeline and IDentification. Available at: <https://doi.org/10.5281/zenodo.7829342>.

3. Hadfield J, Megill C, Bell SM, et al. Nextstrain: real-time tracking of pathogen evolution. Bioinformatics **2018**; 34:4121-3.

4. O'Toole A, Scher E, Underwood A, et al. Assignment of epidemiological lineages in an emerging pandemic using the pangolin tool. Virus Evol **2021**; 7:veab064.

**Statistical Analysis Plan for “Effect of Omicron-based Compared to Prototype One-Dose mRNA Vaccination on Incidence of COVID-19 in the COVAIL Trial”**

1. **Introduction**

COVAIL enrolled approximately 1250 adults in the U.S. previously vaccinated with a COVID-19 vaccine primary series and one booster. Participants received a homologous or heterologous/variant boost (second booster) from March 30 to October 28, 2022: Stage 1 was enrolled between 30 March to 06 May 2022; Stage 2 was enrolled from 09 May to 27 May 2022; Stage 3 was enrolled from 06 June to 17 June, 2022; and Stage 4 was enrolled from 04 October to 28 October, 2022. The randomization was stratified by age and history of confirmed prior SARS-CoV-2 infection, with the goal of approximately 45% of participants in each arm to include older adult (≥ 65 years of age) and at least 20% with prior infection history. Participants were randomized to one of 17 vaccine booster arms in four stages: Stage 1 is 6 Moderna mRNA vaccine arms (n=600 planned), Stage 2 is 6 Pfizer mRNA vaccine arms (n=300 planned), Stage 3 is 3 Sanofi recombinant protein vaccine arms (n=150 planned), and Stage 4 is 2 Pfizer bivalent mRNA vaccine arms (n=200 planned). Each of the first three stages includes an Ancestral strain prototype vaccine with the remainder of vaccine arms being variant vaccines. All study arms deliver a single booster except vaccine arm 3 in Stage 1 that delivers two bivalent mRNA boosters.

Table 1 shows the COVAIL study schema.

**SAP Table 1: COVAIL study schema**

1. This SAP restricts to the description and comparison of incidence of COVID-19 between participants who received a single Omicron-based booster vs. those who received a single Prototype-based booster in each of Stage 1 and 2 separately. **Antibody markers and sampling design for measuring antibody markers**

Pseudovirus 50% serum inhibition neutralization antibody titers (ID50) were measured against D614G, Delta/B.1.617.2, Beta/B.1.351, Omicron BA.1/B.1.1.529, and Omicron BA.4/BA.5 at multiple timepoints: baseline/enrollment (D1), 14 days after vaccination (D15), 28 days after vaccination (D29) 90 days (D91) after vaccination, 180 days (D181) after vaccination, 270 days (D271) after vaccination, and 365 days (D366) after vaccination, where Omicron BA.4/BA.5 was only measured in a subset at D15. A central lab (Monogram) measured the nAb titers using a validated pseudovirus neutralization assay. ID50 readouts are in arbitrary units/ml (AU/ml). For the D614G strain, AU/ml can be translated to IU50/ml units (calibrated to the 20/136 WHO International Standard) by multiplying AU/ml readouts by 0.0653, as previously described (Fong et al., 2022). For the 4 other strains, there is no International Standard, such that IU50/ml units do not exist. Neutralization nAb ID50 titer against BA.4/BA.5 was measured for all participants at D29 and for a subset at D15. In contrast nAb ID50 titers against the other 4 strains was measured for all participants at D15. In addition, a maximal signal diversity weighted (MDW) score, calculated as the maximal signal diversity weighted average of the log10 ID50 markers against the individual strains (*He and Fong, 2019*).

1. **Vaccine groups of interest for studying vaccine relative efficacy within each Stage**

Because randomization was only performed within each stage, and follow-up was not completely contemporaneous for Stage 1 and 2, all comparative analyses of the incidence of COVID-19 will be restricted to randomized vaccine arms within each stage:

- 1. [Stage 1 (Moderna) Prototype vs Stage 1 (Moderna) Omicron-Containing restricting to one-dose arms] Stage 1 Prototype vaccine (arm 1) vs. Stage 1 Omicron-Containing vaccines (arms 2, 4, 5, 6 pooled)
  2. [Stage 2 (Pfizer) Prototype vs Stage 2 (Pfizer) Omicron-Containing] Stage 1 Prototype vaccine (arm 7) vs. Stage 1 Omicron-Containing vaccines (arms 8, 9, 12 pooled)

1. **Endpoint definition; study timeframe; study cohort**

In this analysis, the COVID-19 endpoint was the symptomatic subset of self-reported or study-conducted positive SARS-CoV-2 tests, the first occurrence of such an event.

For implementing the analysis/computer code, the following time periods for counting COVID-19, and for defining time-to-event variables on the study time scale, are defined using the following rules:

- 1. Early infection: An endpoint is defined as an early infection if it occurs prior to 7 days post D15 visit.
  2. D15_7to91 (Booster-proximal) COVID-19: An endpoint is defined as a booster-proximal endpoint if it occurs between 7 days post D15 visit through 91 days post D15 visit.
  3. D15_92to188 (Booster-distal) COVID-19: An endpoint is defined as a booster-distal endpoint if it occurs between 92 days post D15 visit through 188 days post D15 visit, where 188 days is selected instead of 181 days to include more COVID-19 cases through ~6 months post marker measurement accounting for visit window variability.

Above categorization of COVID-19 endpoint is exhaustive, meaning that each COVID-19 endpoint that occurs since D1 visit through 188 days post D15 visit belongs to one and only one category above.

Participant follow-up is right-censored by the first event among (1) receipt of a second dose (this would only be applicable for study arm 3, but study arm 3 is not included in this project that excludes the two-booster arm), (2) early termination, (3) receiving an out-of-study boost, and (4) the data cut date of 2023-07-13.

To be included in the current analyses a participant must not have an eligibility deviation (based on the variable eligibility_deviation in the data set) and they must have available D15 nAb ID50 titer data.

1. **Descriptive statistics and comparison of baseline covariates and nAb ID50 titers**

Baseline covariates in each of the Prototype and Omicron-Containing arms will be summarized in each stage. Balance will be assessed using a Student’s t test for continuous variables and chi-squared test for categorical variables. The log10-scale nAb ID50 titers at each timepoint will be summarized using mean and 95% CI (based on one-sample t-test) and plotted separately for the Prototype and Omicron-Containing arms in each stage.

Baseline demographics in the Prototype or Omicron-Containing arm between Stage 1 and Stage 2 will be compared using a Student’s t test (continuous variable) and chi-squared test (categorical variable). Baseline nAb ID50 titers and the MDW score will be compared between Stage 1 and Stage 2 adjusting for baseline demographic variables including age, sex assigned at birth, naïve/non-naïve status and the baseline risk score.

1. **Cumulative incidence of COVID-19**

For each of the two-group comparisons listed in Section 3 (Prototype vs Omicron-Containing in Stage 1; Prototype vs Omicron-Containing in Stage 2), for each Prototype or Omicron-Containing arm, the cumulative incidence of COVID-19 over time starting 1 day after enrollment through to 188 days post D15 visit will be plotted. The cumulative incidence curves are estimated using a method described in Westling et al. (2024) and implemented in the R packages CFsurvival (<https://github.com/tedwestling/CFsurvival>) and survSuperLearner (https://github.com/tedwestling/ survSuperLearner). The method provides a way to adjust for baseline covariates and to allow for covariate-dependent right-censoring. A baseline covariate adjusted for is an exogenous force of infection (FOI) score defined based on a data base of COVID-19 incidence in the U.S. during the period of COVAIL follow-up, as noted in Section 9. In addition, the cumulative incidence analysis adjusts for a risk score built via super-learning as described in Section 9 and a participant’s baseline naïve/non-naïve status, where naïve/non-naïve status is coded by the variable ***Infstat*** (data set received from Emmes) that includes both anti-N serotesting data at enrollment and self-report.

Estimating the covariate-adjusted cumulative incidence curves involve estimating nuisance parameters. Super-learner is used to generate estimates of the propensity score, conditional censoring distribution and to generate initial estimates of the conditional COVID-19 outcome regression. The Super Learner library includes both parametric and nonparametric algorithms as specified in Table 2. If a candidate algorithm involves tuning parameters, then the tuning parameter is selected based on a 5-fold cross validation.

**SAP Table 2. Super Learner library of regression models for estimation of the conditional survival function of COVID-19 event, conditional survival function of censoring, and the propensity score for TMLE estimation of the counterfactual cumulative incidence of the COVID-19 endpoint**

| **Conditional survival function of COVID event** |  |
| --- | --- |
| survSL.km | Kaplan-Meier estimator |
| survSL.cox | Cox model |
| survSL.rfsrc | Survival random forest |
| survSL.gam | Generalized additive model |
| **Conditional survival function of censoring** |  |
| survSL.km | Kaplan-Meier estimator |
| survSL.cox | Cox model |
| survSL.rfsrc | Survival random forest |
| survSL.gam | Generalized additive model |
| **Propensity score** |  |
| SL.mean | Mean |
| SL.glm | Generalized linear model |

1. **Cumulative incidence ratio analysis**

Cumulative incidence ratios over time [CIRs(t)] will be estimated by the ratio (Prototype/Omicron-Containing) of the cumulative incidence estimators described in Section 6. Influence-curve based variance estimators of each cumulative incidence is used, and the delta method applied to obtain the variance estimator of the log cumulative incidence ratio. Point estimates and 95% pointwise transformed Wald CIs for cumulative incidence curves and CIR(t) curves will be plotted. Note that while COVID-19 endpoints from D1 are included in the cumulative incidence curves, COVID-19 endpoints prior to 7 days post D15 (referred to as early infections in Section 4) are not included in the cumulative incidence ratio estimates, so that the study cohort in such comparative analyses is consistent with those studied for the evaluation of immune correlates.

1. **Cox regression analysis**

To summarize overall vaccine effects, the COVID-19 hazard ratio comparing Prototype and Omicron-Containing vaccines will be reported as a point estimate, 95% CI, and 2-sided *P*-value for whether the hazard ratio departs from unity. Similar to the cumulative incidence ratio analysis, Cox regression analysis excludes COVID-19 endpoints prior to 7 days post D15. Cox regression analysis will be conducted for the booster-proximal period (7 days post D15 visit through 91 days post D15 visit) and booster-distal period (92 days post D15 visit through 188 days post D15 visit) combined and for each period separately.

The analysis is done with a proportional hazards model using calendar time as the time scale (thus allowing flexible modeling of COVID-19 incidence over calendar time in the nonparametric estimation of the baseline hazard function), with time origin the first enrollment in each stage. These analyses include separate baseline hazards for naïve and non-naïve enrolled participants. The analysis adjusts for the baseline risk score but not the FOI score (unlike the cumulative incidence analyses) because Cox regression analyses are calendar-time-based. The Cox regression analysis will also be repeated among baseline naïve, baseline non-naïve, and baseline anti-N positive participants.

Hypothesis tests for goodness of proportional hazards model fit will be conducted using the Grambsch and Therneau (1994) method as implemented in the R function cox.zph in the survival library.

1. **Baseline risk score and force of infection (FOI) score development**

A baseline risk score for best predicting occurrence of COVID-19 starting 7 days after the D15 visit will be built using cross-validated super learning pooling over all study participants over all four stages, based on all demographic and vaccination history input variables, using a similar approach as taken for assessing immune correlates in the phase 3 trials through the US Government’s COVID-19 Vaccine Correlates of Protection Program. In particular, the baseline risk score is defined as the logit of the predicted COVID-19 outcome probability from a regression model estimated using the ensemble algorithm superlearner (i.e. stacking), where this logit predicted outcome is scaled to have empirical mean zero and empirical standard deviation one. The settings of superlearner (i.e., loss function, cross-validation technique, library of learners) that are used for implementation of superlearner for building a baseline risk score are specified as follows:

The binary endpoint implementation of superlearner is used without accounting for right-censoring, with the needed details recapitulated as follows:

1. Pre-scale each quantitative and ordinal input variable to have empirical mean 0 and standard deviation 1.
2. Negative binomial log-likelihood loss is used.
3. Study participants lost to follow-up without ever experiencing a COVID-19 endpoint are excluded from the analysis, for the relevant time-period of analysis.
4. For analyses including all of follow-up, 5-fold cross-validation is used, with no more than floor(nv/6) input variables included in any given model/algorithm where nv is the number of evaluable COVID-19 endpoint cases.
5. Learning algorithms (listed in Table 2) are included with and without screening of variables. Screens used will be: 1) glmnet (lasso) pre-screening (with default tuning parameter selection), 2) logistic regression univariate 2-sided p-value screening (at level p < 0.10), and 3) high-correlation variable screening (described below).
6. Include high-correlation variable screening, not allowing any pair of input variables to have Spearman rank correlation r > 0.9.
7. The superlearner is conducted averaging over 10 random seeds, to make results less dependent on random number generator seed.
8. Inverse probability of sampling (IPS) weights are not needed, given the measurement of PsV ID50 titer from all participants. If analyses include markers only measured in a participant subset, then IPS weights will be used.
9. Discrete-SL estimated models, derived using the learning algorithms specified in Table 2, will be used to compare the relative performance for each of the variable sets based off the estimated cross-validated area under the ROC curve (CV-AUC) with a 95% confidence interval.
10. Two levels of cross-validation are used:
    1. Outer level: CV-AUC computed over 5-fold cross-validation repeated 10 times to improve stability
    2. Inner level: 5-fold CV used to estimate ensemble weights, unless the number of COVID-19 endpoints is less than 50 in which case leave-one-out CV is used.
11. Classification accuracy is summarized by point and 95% confidence interval estimates of CV-AUC (*Hubbard, Pajouh, and van der Laan, 2016; Williamson et al., 2021*). CV-AUC is estimated using the R package vimp available on CRAN.

The following baseline demographic input variables were included for building the risk scores: age in years, indicator of age ≥ 65, sex assigned at birth (Male/Female), ethnicity, race, the number of days from last prior vaccination until enrollment, the indicator that the number of days from last prior vaccination until enrollment is greater than the median value, and the type of last vaccine received prior to the booster.

The development of the baseline risk score involves training the superlearner including study participants from all 17 arms in the COVAIL study and making cross-validated (CV)-predictions. First-occurrence COVID-19 endpoints are included and counted starting 7 days post D15, with all COVID-19 endpoints counted ignoring lineage information. The CV-prediction performance of superlearner is characterized by point and 95% confidence interval estimates of the cross-validated area under the ROC curve (CV-AUC) and by point estimates of CV-ROC curves, which are calculated based on first occurrence COVID-19 endpoints starting 7 days post D15. The baseline risk score is defined as the logit of the predicted outcome probability from the superlearner model, where this logit predicted outcome is scaled to have empirical mean zero and empirical standard deviation one.

The baseline risk score, as long as it has some predictive capacity (defined as CV-AUC > 0.55), is adjusted for in all analyses. SARS-CoV-2 naïve/non-naïve status, vaccination type information, and D1 ID50 marker data are not included as input variables into the baseline risk score, as they are treated in a special way for various correlates analyses.

In addition, the cumulative incidence analyses also adjust for a force of infection score (FOI score) calculated with data from the Coronavirus Resource Center’s database hosted by Johns Hopkins University (JHU): [United States - COVID-19 Overview - Johns Hopkins (jhu.edu)](https://coronavirus.jhu.edu/region/united-states). This database provides COVID-19 case numbers and incidence rates in the U.S. from 23 January 2020 through 09 March 2023. Each study participant’s FOI score is computed as the average of daily COVID-19 incidence rates (number of cases per 100,000 persons) over all days spanning from 7 days after their D15 visit through to 188 days post enrollment. The JHU database contains data at the geographic level of U.S. state and some territories, so the participants’ geographic locations (at the level of state or the District of Columbia) were factored into the calculation.

FOI scores were not calculated for participants who missed their D15 visit, which does not impact correlates analyses given that D15 visit attendance is required for inclusion. Additionally, a small number of participants had follow-up periods that extended beyond the JHU database’s final day of 09 March 2023 (the latest 188 days post booster date for a COVAIL participant was 04 May 2023). The case numbers for these missing dates were extrapolated by using the case numbers for those days from the prior year (10 March 2022 through 04 May 2022). In almost all geographic regions, these extrapolations were comparable with the preceding observed case counts, suggesting that they are a reasonably good fit. The final FOI score used for covariate adjustment is standardized to have empirical mean 0 and empirical standard deviation 1.

1. **Multiple hypothesis testing adjustment**

All p-values are 2-sided and no adjustments for multiple hypothesis testing are performed.

***SAP References:***

Fong Y, McDermott B, Benkeser D, et al. on behalf of the Immune Assays Team, the Janssen Team, the Coronavirus Vaccine Prevention Network (CoVPN)/ENSEMBLE Team, and the United States Government (USG)/CoVPN Biostatistics Team. Immune correlates analysis of a single Ad26.COV2.S dose in the ENSEMBLE COVID-19 vaccine efficacy clinical trial. *Nature Microbiology* 2022: 1-15.

Grambsch PM, Therneau TM. Proportional hazards tests and diagnostics based on weighted residuals. *Biometrika* 1994 Sep 1;81(3):515-26.

He Z, Fong Y. Maximum diversity weighting for biomarkers with application in HIV-1 vaccine studies. *Statistics in Medicine* 2019; 38(20): 3936-3946.

Hubbard AE, Kherad-Pajouh S, van der Laan MJ. Statistical inference for data adaptive target parameters. *The International Journal of Biostatistics* 2016 May 1;12(1):3-19.

Williamson BD, Gilbert PB, Simon NR, Carone M. A general framework for inference on algorithm-agnostic variable importance. *Journal of the American Statistical Association* 2021 Dec 22:1-14.

Westling T, Luedtke A, Gilbert PB, Carone M. Inference for treatment-specific survival curves using machine learning. *Journal of the American Statistical Association* 2024 Apr 2;119(546):1541-53.
